# Quality indicators for the referral process from primary to specialised mental health care: an explorative study in accordance with the RAND appropriateness method

**DOI:** 10.1186/s12913-016-1941-1

**Published:** 2017-01-03

**Authors:** Miriam Hartveit, Kris Vanhaecht, Olav Thorsen, Eva Biringer, Kjell Haug, Aslak Aslaksen

**Affiliations:** 1 Research Network on Integrated Health Care in Western Norway, Helse Fonna Local Health Authority, Helse Fonna HF, Box 2170, 5504, Haugesund, Norway; 2 Department of Global Public Health and Primary Care, Faculty of Medicine and Dentistry, University of Bergen, Bergen, Norway; 3 School of Public Health, KU Leuven, University of Leuven, Leuven, Belgium; 4 Department of quality management, University Hospitals Leuven, Leuven, Belgium; 5 Leuven Institute for Healthcare Policy, School of Public Health, University of Leuven, Leuven, Belgium; 6 Department of Radiology, Haukeland University Hospital, Bergen, Norway

**Keywords:** Referral and consultation, Quality of health care, Quality indicators, health care, Process assessment (health care), Mental health services, RAND appropriateness method

## Abstract

**Background:**

Communication between involved parties is essential to ensure coordinated and safe health care delivery. However, existing literature reveals that the information relayed in the referral process is seen as insufficient by the receivers. It is unknown how this insufficiency affects the quality of care, and valid performance measures to explore it are lacking. The aim of the present study was to develop quality indicators to detect the impact that the quality of referral letters from primary care to specialised mental health care has on the quality of mental health services.

**Methods:**

Using a modified version of the RAND/UCLA appropriateness method, a systematic literature review and focus group interviews were conducted to define quality indicators for mental health care expected to be affected by the quality of referral information. Focus group participants included psychiatrists, psychologists, general practitioners, patient representatives and managers. The existing evidence and suggested indicators were presented to expert panels, who assessed the indicators by their validity, reliability, sensitivity and feasibility.

**Results:**

Sixteen preliminary indicators emerged during the focus group interviews and literature review. The expert panels recommended four of the 16 indicators. The recommended indicators measure a) timely access, b) delay in the process of assessing the referral, c) delay in the onset of care and d) the appropriateness of the referral. Adjustment was necessary for five other indicators, and seven indicators were rejected because of expected confounding factors reducing their validity and sensitivity.

**Conclusions:**

The quality of information relayed in the referral process from primary care to specialised mental health care is expected to affect a wide range of dimensions defining high quality care. The expected importance of the referral process for ensuring ‘timely access’-one of the six aims of high-quality health care defined by the Institute of Medicine-is highlighted. Exploring the underlying mechanisms for the potential impact of referral information on patient outcomes is recommended to enhance quality of care.

**Trial registration:**

ClinicalTrials.gov: NCT01374035 (28 April 2011).

## Background

Information provided in the referral process constitutes the main communication from primary care to specialist health care [1, 2]. Existing literature reveals, however, that the information relayed in the referral process is seen as insufficient by the receivers [1–4]. The transit from primary care to specialist health care constitutes a major clinical handover situation, implying a large risk of adverse events [5, 6]. Further, coordination across services is one of the major challenges to health care [7]. Improving the information transference is the principal means of reducing the risk of adverse events in clinical handovers and ensuring continuity and coordination of care [5, 6, 8–10]. Mental health care is often provided by various primary health and social services, in combination with periods of specialised mental health care [11]. Patients with mental illnesses are therefore particularly vulnerable to the effects of insufficient referral information. Nevertheless, there is a striking lack of research on whether and how the quality of referral information affects the quality of care [1, 12].

To explore the impact of referral information on quality of care, as well as the underlying mechanisms through which this effect may be realised, it is necessary to establish valid measurements for the output of the referral process [13]. The quality of the referral process can be assessed on three dimensions: necessity (whether the patient should be referred), destination (where the patient should be referred) and quality [14]. The ‘quality’ dimension concerns the process of referral, in which the quality of the referral letter is essential [14, 15]. Sufficient information is the most essential criterion for assessing the quality of the referral letter; most of the existing literature on referral letters’ quality and interventions to improve this is on the completeness of information relayed in the letter [1, 2, 15]. The construct of ‘high quality referral letter to specialised mental health care’ is therefore often defined by the completeness of information in the letter, as was done by Hartveit et al. [16]. The Quality of Referral information-Mental Health (QRef-MH), a recently developed and tested instrument, provides a valid operationalisation of the construct [17]. The instrument includes 19 items regarding identification of the patient, essential introductory information (included as check-off points), case history and social situation, present state and results, past and on-going treatment efforts and involved professional network, the patient’s assessment, and reason for the referral [17].

Existing literature reveals a large set of outcome indicators relevant for exploring the quality of health care, including readmission rate, mortality and patient experiences measured through surveys [18]. Indicators can be defined as ‘measures that assess a particular health care process or outcome’ [19]. They should be valid and reliable, sensitive to change, acceptable, feasible and easy to communicate [13, 19]. Indicators are used to assess structures, processes and outcomes in health care [19]. Existing outcome measures do not enable us to understand how and why referral information may affect the quality of care. It has therefore been recommended to develop indicators for sub-processes in health care, such as the referral process [12, 19, 20].

Exploring the underlying mechanisms through which referral information may influence quality of care is recommended for several reasons. First, an understanding of the underlying processes linking referral information to quality of care (e.g., mediating factors) will enable us to develop interventions tailored to support these mechanisms [13]. Second, mediating factors can affect a wide range of important outcome measures [13]. Consequently, the detection of such key mediating factors will facilitate the effective improvement of outcomes. Third, the use of indicators measuring mediating factors will make possible the identification of improvement potential and evaluation of improvement efforts, because these indicators are more sensitive to change than are outcome measures [20]. In the complex intervention of a care pathway (a systematic method to improve care across different patient groups), which is found to be effective in improving coordination and communication in health care processes, indicators serve an essential role in the improvement process [21]. For research purposes, revealing mediating factors is essential for developing theories of causality and exploring to what degree changes in these factors predict improved patient outcomes [22, 23]. The thorough development of valid process and outcome indicators is supported by the guidelines of the United Kingdom’s Medical Research Council ((UK) MRC) for exploring the causality and predictive value of a complex intervention on relevant outcomes [24]. Theory and evidence derived through research exploring components in complex processes and interventions will enable the informed use of theory in improvement programmes, as recommended by Davidoff and colleagues [25]. For mental and substance use health care, the development of indicators is particularly recommended, because few measures have been developed and the improvement infrastructure within these services suffers from limitations [11]. This is also true for the referral process, where the evidence for valid indicators to detect the mechanisms and effects of improved referral is clearly limited [1].

The present study’s aim was to develop quality indicators measuring the impact of referral information from primary care to specialised mental health care to explore how the quality of this information can affect the quality of mental health care for adults. The construct of ‘referral information’ was defined in accordance with the guidelines established by Hartveit et al. [16] and operationalised using QRef-MH [17].

## Methods

The study was conducted in the region of the Western Norway Regional Health Authority, which is responsible for public specialised health care for a population of approximately one million. In response to the research question ‘What indicators are relevant and valid in the assessment of the potential impact of improved referral information on specialised mental health care for adults?’, we adapted the RAND/UCLA appropriateness method [26, 27] and used a stepwise process as described in Table 1. First, we organised focus group interviews with participants representing the most central stakeholders. Second, we conducted a systematic literature review. Finally, indicators identified in the focus group interviews and the literature review were assessed using criteria for indicators (see Table 2) by expert panels [13, 27]. The RAND/UCLA appropriateness method was chosen for its strengths in combining the best available evidence and collective judgement by experts to assess and select indicators in areas with limited existing knowledge, as is the case for the referral process [26]. To enrich the material and gain a deeper insight into areas of mental health care potentially affected by referral information, the method was supplemented by focus groups interviews.

**Table 1:** The steps in the RAND/UCLA appropriateness method and the present study

The steps in the RAND/UCLA appropriateness method [26, 27]	The steps in the present study
	Focus group interviews including patient representatives, managers and health professionals
Systematic literature review	Systematic literature review
Generate preliminary indicators	Preliminary indicators generated from both focus group interviews and literature review
Selection of expert panel	Selection of experienced specialists and researchers in specialised mental health care
Presentation of existing evidence and individual rating (postal)	Panel meeting with oral presentation of existing evidence with opportunity for individual reflections before discussion and assessment of the preliminary indicators
Panel meeting with presentation of the first rating, discussion and assessment of the preliminary indicators	
Analysis of final rating	The groups’ assessments and categorising of the indicators were analysed by two researchers individually
Development of recommended indicators	Development of a ranked list of indicators

**Table 2:** Criteria for indicators used by the expert panels

Criteria for indicators used by the expert panels [13, 27]	
Validity:	The extent to which the indicator accurately represents the concept being assessed
Reliability:	The degree of trustworthiness of the data collected by the indicator
Sensitivity to change:	The degree to which the indicator is affected by change in the quality of referral letters
Acceptability:	The degree to which stakeholders find the indicator relevant
Feasibility:	The extent to which it is possible to gather data within defined frames such as economic, legal and time constraints
Simple and communicable:	The degree to which the results are easy to communicate and understood by the intended audience

### Focus group interviews

Four focus group interviews [28] were conducted to define quality indicators or areas expected to be affected by improved referral information. To stimulate discussion and gain insight into the subject from different perspectives [28], each focus group was composited by health professionals, patient representatives and managers. Nine focus group participants worked in primary or specialised health care, six were managers and four were patient representatives. Of the 15 participants representing the professional and management perspective, nine were medical doctors (two general practitioners), four were psychologists and two were nurses. Twelve of these were specialists. Three of the four patient representatives had more than 15 years of experience with mental health care. The participants were selected by their organisations in the region because of their interest in and knowledge of the topic.

At the beginning of the group interviews, the participants discussed what type of information they recommended including in a specialised mental health care referral request. (These findings have been published separately [16]) After the discussion, they were asked, ‘If the referral letters were improved in the way you suggest, how do you think this would affect the process of care?’ The interviews were structured using the ‘affinity diagram’ [29], which included steps for written brainstorming using post-it notes. This method ensures a common understanding of ideas among the group members and excludes overlapping ideas [29]. The brainstorming was conducted in two sessions, with an oral discussion in between. The interviews were moderated by a researcher (EB) and observed by a second researcher (MH). All interviews, where the participants explain their ideas, were audio recorded to provide additional information for the analyses.

The suggested ideas (written by the participants on post-it notes) were analysed by two researchers (MH and OT) individually, guided by the steps of systematic text condensation by Giorgi, as described by Malterud [30]. Both researchers read all of the notes and listened to the audiotape to clarify the content of the notes to gain an overview, and the notes were then categorised by similarities in content and a code was defined for each category. For each category, the emerging indicators were defined. Finally, the results of the individual analyses were discussed by the two researchers, and a consensus about categories and preliminary indicators was reached.

### Literature review

The literature search was conducted using PsycINFO, Embase and PubMed over a period of 10 years (2002–week 26 in 2012). The scarcity of existing literature necessitated wide inclusion criteria: All papers revealing, suggesting or discussing a potential causal chain between contents of referral information and aspects of quality of care were included. However, articles suggesting indicators clearly relevant for only one mental health diagnosis were excluded as ‘diagnosis-specific’. The search was conducted in the three databases for articles where the phrase ‘referral letter(s)’ occurred in the title or in the abstract, and was limited to adult patients. Based on the abstracts, articles were selected for full text reading, and relevant preliminary indicators were identified. Two authors (MH and OT) discussed and reached a consensus on the combined results from the interviews and the systematic literature review.

### Expert panels

Three expert panels were set up, with three, three and two participants. The participants were all experienced psychiatrists or trained psychologists, and four were also experienced researchers. They were asked to assess each indicator using criteria for indicators regarding validity, reliability, sensitivity to change, acceptability, feasibility, simplicity and communicability [13, 19, 27]. The criteria, as introduced to the panels, are described in Table 2.

The aim of the study was described to the panels before they were presented with the indicators and their evidence basis, which was derived from the focus groups and literature review. Indicators were first evaluated by the individual members of the panel. The panel then discussed to what degree the indicators met the criteria for good indicators (Table 2). The expert panels were requested to place the indicators in one of three groups: bad/unacceptable, acceptable/needs adjustment or good/recommended. Further, they were encouraged to suggest improvements to the indicators. One researcher (MH) presented information to the panels and moderated the discussion, and two of the three groups also included an observer. At the end of the discussion, the moderator introduced relevant arguments made by the other expert panels and gave the panellists an opportunity to assess the suggested indicator once more to maximise the benefits of conducting multiple panels.

## Results

**Fig. 1:**
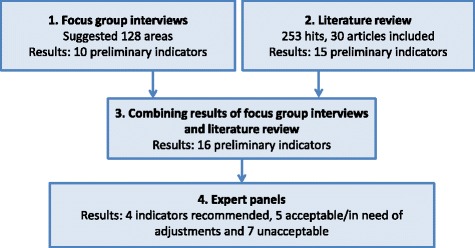
Illustration of the study

The results of each step in the study are shown in Fig. 1.

### Focus group interviews

After excluding intergroup duplicates, the four groups suggested 128 indicators or areas (potential mediating factors) expected to be affected by improved referral information. During the analyses, three categories of suggestions emerged: co-operation, timely access and organisation/logistics. ‘Co-operation’ included suggestions such as a common understanding of and respect for the distribution of responsibility between primary care and specialised health care, avoiding duplication of interventions and improved co-ordination between the involved services. ‘Timely access’ comprised performance measures on improved decision making to ensure that the patients assessed as (medically) most in need receive specialised mental health care first. Most suggestions within ‘organisation/logistics’ concerned delays and waste in the process of care and focused on the optimal use of scarce specialised health care resources, such as the specialists’ time. Ten preliminary indicators emerged from the three categories. Of these 10 indicators, four where in the category of ‘co-operation’, three were in ‘timely access’ and three were in ‘organisation/logistics’.

### Literature review

The literature search resulted in a total of 253 hits (PubMed: 88, PsycINFO: 24 and Embase: 141). Applying the inclusion criteria, 30 articles were included, whereas only three were from the database for mental health, PsycINFO. During the analyses, five categories evolved defining potential areas expected to be affected by the quality of referral information (with reference to the included papers in square brackets): timeliness and delay [31–33], attendance/drop-out [34–37], unnecessary consultations and investigations [32, 38–42], appropriateness of the referral [32, 43–53] and correctness of prioritisation of patients [36, 40, 44, 54–60]. Fifteen preliminary indicators were derived from the abstract of these five categories.

The 15 preliminary indicators suggested by the literature review were fully supported by the areas suggested by the focus group interviews. In addition to these, the focus group participants suggested measuring the degree of common understanding of the treatment plan among the involved services and health professionals. For further specification of the 16 indicators, the research team used their experience in mental health service provision and indicator development and consulted colleagues in the clinic on an ad hoc basis.

### Expert panels

The expert panels’ assessment of the appropriateness of the indicators resulted in the recommendation of four of the 16 suggested indicators (Described in Table 3). The indicator ‘timely access’ measures whether the specialist’s assessment of urgency (maximum acceptable waiting time) based on information given in the referral letter correlates with a corresponding assessment based on a clinical evaluation. Two indicators measuring delay in the process were also among the recommended indicators. The first of these measures was whether the receiver of the referral was immediately able to determine the priority of the patient, or whether he/she had to request further information to prioritise the patient correctly. The second delay in process indicator concerned waiting time to start specialised health care treatment for patients with a severe condition and for patients with a less severe condition. Severity is defined by ‘severity factors’ [16] regarding risk of harming oneself or others, substance abuse, psychosis and caring for children. The fourth recommended indicator is appropriateness of referral. It measures whether the hospital specialist perceives the referral to be timely and to describe a situation where referral is recommended.

**Table 3:** Description of the four recommended indicators

1. TIMELY ACCESS	
Rationale and definitions	To ensure timely access for all referred patients, prioritisation of patients is needed. Priority of a referred patient is determined by a specialist based on severity of the condition and urgency because of social factors. It is defined by maximum (medically) acceptable waiting time (see footnote^a^). Scarce information in referral letters implies a risk of incorrectly assigned priority. A review of the priority based on information from the first consultations can give an indication of the correctness of the first priority decision. Correct priority is defined as equal indications of acceptable waiting time in both instances, with waiting time divided into four categories.
Numerator	Number of referrals where there is a match between the priority given based on the referral letter and the priority seen as correct retrospectively based on 1–3 consultations.
Denominator	Number of all referrals.
Methodological challenges	Long waiting time implies a larger risk for changes in the patient’s mental state. It is therefore recommended to explore the impact of time between the first and second priority-settings. Guidelines for deciding acceptable waiting time must be clearly defined, and a common understanding of these among the specialists is needed.
Possible approaches for quality improvements	There may be disagreement between stakeholders (e.g., the patient, specialists and the GP) on optimal prioritisation of patients and acceptable waiting time. Data on the waiting time that is seen as acceptable by the referring GP and the patient, in addition to the specialists’ assessments, will provide a fuller picture of ‘correct prioritisation of patients’.
2. DELAY IN PROCESS OF ASSESSING THE REFERRAL	
Rationale and definitions	If referral letters lack necessary information, initiatives to collect additional information, such as contacting the referring GP or the patient, can postpone the assessment of the referral. Then, sending a response letter to the patient and the referring GP is correspondingly delayed. High quality referral letters are expected to include enough information for priority setting without delay. Delay in the process of priority is defined as not sending the response letter after the first assessment because of insufficient referral information.
Numerator	Number of response letters sent after first assessment of the referral.
Denominator	Number of all referral letters assessed.
Methodological challenges	Contextual differences may affect the validity (e.g., the tradition for collecting additional information may vary between units).
Possible approaches for quality improvements	If the decision is made individually and not by regular assessment meeting, a more informative indicator, such as mean number of days delayed, can be used.
3. WAITING TIME FOR HIGH PRIORITY PATIENTS	
Rationale and definitions	To select the patients most in need, the present study recommended defining severity according to symptoms or situation rather than diagnosis. Severity can be determined by the existence of a combination of the following severity factors: severe mental illness/psychosis, risk of suicide, risk to others, in care of children, substance abuse and younger than 23 years [57]. High quality referral letters are expected to enable specialists to prioritise the patients most in need to a greater extent than low quality referral letters. ‘High priority patients’ are defined as patients suffering from three or more of the severity factors detected by a specialist after the first 1–3 consultations. Waiting time is defined as days from receipt of the referral letter by the specialised health care to the onset of (specialised) care.
Numerator	Median waiting time for patients with three or more severity factors.
Denominator	Median waiting time of all patients.
Methodological challenges	The cut-off at three severity factors currently lacks empirical support. There is a risk of false positive and false negative findings, as the presence of three severity factors does not always indicate a greater severity than the presence of two factors.
Possible improvements of the indicator	It is recommended to explore whether three is the most appropriate cut-off for the severity factors to define patients who should have less waiting time. Further, exploration of each factor’s impact can reveal whether the factors should be weighted to reduce the risk of false negative or false positive findings.
4. APPROPRIATENESS OF REFERRAL	
Rationale and definitions	High quality referral letters include information about necessary tests, examinations and treatment efforts that were conducted prior to the referral. The quality of referral letters is therefore expected to be positively correlated with the appropriateness of the referral. ‘Appropriate referral’ is defined as referrals assessed by the receiving specialist as appropriate on a dichotomous variable (Yes/No).
Numerator	Number of appropriate referrals.
Denominator	Number of all referrals.
Methodological challenges	The sensitivity to change is limited for dichotomous variables. There might be disagreement between primary care and specialised mental health care with regard to appropriateness. This indicator represents only the specialist health care provider’s perspective of appropriateness of referrals.
Possible improvements of the indicator	The reliability of an ordinal variable should be tested. The potential disagreement between service providers on the appropriateness of a referral can be explored.

^a^According to the legal rights for patients in Norway, all patients referred to specialised health care are prioritised by a specialist. High priority which entails a legal right to health care with a (medically) defined deadline for when health care should be provided; low priority, which means the patient will receive health care, but there is no guarantee as to when it will be provided; or no priority, which means the patient is not in need of specialised mental health care. The assessment is usually done on the basis of the referral letter, but more information can be gathered

In all expert panels, participants spontaneously expressed that they saw the quality of referral information as a factor important for the quality of health care. However, they were also explicit about the difficulties they saw with defining good indicators according to the defined criteria [13, 27]. Seven of the 16 indicators presented were assessed as unacceptable by all three panels or as unacceptable by two and ‘acceptable/in need of adjustments’ by the third panel. The panellists saw the suggested causal chain as clearly weak or questionable because of a large expected risk of confounding factors affecting these seven indicators. Further, limited feasibility was given as a counterargument for some of the indicators. Five indicators were seen as acceptable or in need of improvements by all panels or by two and as unacceptable by the third. The participants expressed that they expected these indicators to represent existing causal chains but were in doubt as to the strength of the causal chains, strength of confounding factors and/or reliability. The 12 indicators that were not recommended, i.e., found to be in need of adjustments or to be unacceptable, are described in Table 4.

**Table 4:** Description of the 12 indicators that were not recommended

	Rationale and definitions	Numerator Denominator	Methodological challenges and possible improvements
INDICATORS FOUND ACCEPTABLE/IN NEED OF ADJUSTMENTS			
1. Rejected referrals	Insufficient referral information makes the specialists less confident in their decisions on whether the referral request should be rejected or not. High quality referral letters can better enable specialists to reject patients not in need of specialised mental health care (instead of seeing them to be “on the safe side”) than can those of low quality. Rejected referral is defined as referral assessed by a specialist as not meeting the criteria for receiving specialised mental health care.	No. rejected referrals No. referrals (total)	Different potential confounding factors. We lack a definition of the optimal number of rejected referrals. Calibration of what the goal should be within different health care systems is needed. A careful exploration to ensure that the rejected referrals are the right ones is essential.
2. Aborted episodes of care	Less informative referral letters can result in incorrect access to specialised health care. This is often detected during the first consultations and the patient is then discharged. Aborted episode of care is defined as terminated by the service after ≤ 3 consultations because of incorrect access to specialised mental health care.	No. episodes of care aborted after ≤ 3 consultations No. episodes of care started	Risks of false positive findings as some episodes of care are completed in 3 or fewer consultations.
3. Severity in high priority patient group^a^ (severity factors)	High quality referral letters can enable specialists to prioritise patients most in need, as defined by the existence of several ‘severity factors’. ‘Severity factors’ are defined as severe mental illness, risk of suicide, risk to others, care for children, substance abuse and being younger than 23 years^b^.	No. patients with 3 or more severity factors in the high priority group No. all patients in high priority group	Risk of both false positive and false negative findings as the existence of 3 factors does not necessarily indicate a larger severity than 2 factors.
4. Realism in expectation toward specialised mental health care	The realism of expectations toward specialised health care formulated in the referral letter, as assessed by the receiving specialist, can be an indicator for the common understanding of the responsibilities of various services. Degree of realism is assigned a score (0–3).	No. letters with score 2 or 3 No. all referral letters	Uncertainty regarding the causal chain. Some of the present letters do not specify expectations (= missing data).
5. Supportive information gathering	Different initiatives by the specialist to gather additional information are needed when referral letters do not convey the information necessary to decide if and when specialised health care should be conducted. Supportive information gathering is defined as extra activities, such as contacting the referring GP or the patient, conducted by the specialised health care because of insufficient information in the referral letter.	No. activities No. referral letters	Contextual variation in the tradition of collecting additional information is a confounding factor. Very high or very low results should be interpreted with caution. A qualitative exploration of the specialists’ reasons for collecting (or not collecting) additional information is recommended when initiatives are almost always or never taken.
INDICATORS FOUND UNACCEPTABLE			
6. Severity in high priority patient group^a^ (diagnosis)	High quality referral letters can better enable specialists to select the patients most in need than can referral letters of low quality.	No. patients with diagnosis of severe illness No. all patients	The diagnosis is not a valid indicator for the degree of need for specialised mental health care.
7. Common understanding of the coordinated care plan	High quality referral letters may facilitate a common understanding of the overall plan for the coordinated care among the involved service providers. A survey where involved professionals tick off the interventions/services they think are involved in each patient’s care plan will reveal the degree of common understanding.	No. plans with a high degree of agreement No. all plans	The integrated plan is not usually defined on the basis of the referral information. Low feasibility.
8. Adequate specialist response (referring GP)	High quality referral letters include a well-defined request that can better facilitate an adequate response than can low quality referral letters. Adequate response is defined as 2 or 3 on an ordinal scale from 0 to 3, assessed by the referring GP.	No. letters with score 2 or 3 No. all referral letters	The response depends on factors in addition to the GP’s request, reducing the validity. Many referral letters do not include a concrete, explicit request, negatively affecting the feasibility.
9. Adequate specialist response (patient)	High quality referral letters include a well-defined request that can better facilitate an adequate response than can low quality referral letters. Adequate response is defined as 2 or 3 on an ordinal scale from 0 to 3 on the adequacy of the specialised health care response assessed by the referred patient.	No. letters with score 2 or 3 No. all referral letters	As for indicator 8. Patient involvement in defining the referral letter is often limited (i.e., the patient is seldom fully aware of, or may not fully agree to, the formulated request), reducing the validity.
10. Time to decide priority	Specialists are expected to spend less time assessing high quality referral letters than low quality referral letters. Time is defined as minutes used for assessing the referral letter including time for gathering extra information.	Minutes to decide priority No. referral letters	The decision is often made step by step including individual assessment and interdisciplinary discussion in the team, negatively affecting the feasibility. Long letters can be of high quality but take more time to read.
11. Attendance to first consultation	Informative referral letters can enable facilitation of the first consultation to the patient’s needs, reducing the risk of non-attendance.	No. non-attending patients No. all patients for first consultation	Several confounding factors expected. Limited sensitivity.
12. Attendance to consultations in first 3 months	High quality referral letters can be associated with less drop-out in the first 3 months of treatment by enabling facilitation, compared with low quality referral letters.	No. drop-out in first 3 months No. all patients completing 3 months of treatment	As for indicator 11. Facilitation is usually based on information provided by the patient rather than by the referral letter.

^a^According to the legal rights for patients in Norway, all patients referred to specialised health care are prioritised by a specialist and given high priority, which entails a legal right to health care with a (medically) defined deadline for when health care should take place; low priority, which means the patient will receive health care, but there is no guarantee as to when it will be conducted; or no priority, which means the patient is not in need of specialised mental health care. The assessment is usually done on the basis of the referral letter, but more information can be gathered

^b^In the Norwegian health care system, patients under 23 years old with a substance abuse problem are, by law, given priority when referred to specialised mental health care

The focus group interviews and expert panels revealed local factors that may affect the indicators’ validity and reliability for benchmarking, such as how the assessment of referral letters is organised and the capacity of the various specialised mental health units. Further, it was emphasised that diagnosis is not seen as an appropriate way to define the degree of patients’ needs or severity of condition and should be replaced by ‘severity factors’, as suggested in a previous study [16]. For all indicators, including those recommended, the expert panels emphasised the need for further development by exploring which factors should be controlled for and testing these factors.

## Discussion

Using a modified version of the RAND/UCLA appropriateness method, the present study explored underlying mechanisms for the potential impact of referral information on the quality of care by responding to the research question, ‘What indicators are relevant and valid in the assessment of the potential impact of improved referral information on specialised mental health care for adults?’ The construct of ‘referral information’ was defined by the inclusion of recommended content in referral letters to specialised mental health care, as described by Hartveit and colleagues [16, 17]. The present study revealed a set of 16 indicators measuring the potential impact of the quality of primary care referral letters on quality of care. Of the identified indicators, four were recommended for use, and five were seen as having potential but in need of further adjustments.

### Results discussed in light of existing literature

Guevara and colleagues have developed a model for the specialty referral process that suggests that the impact of the referral process can be measured within the areas of coordination, resource use, quality and outcomes [12]. The indicators suggested by the present study are in accordance with the model by Guevara and colleagues: Indicators regarding delay and waste of time in the process of handling the referral request translate as ‘resource use’ and ‘coordination’. Indicators of co-operation and timely access regard elements of ‘co-operation’ and ‘quality’ in the model of Guevara and colleagues (i.e., equity, timeliness, appropriateness and integration of care). Further, the results are supported by the Institute of Medicine (IOM), which defines being ‘timely’ as one of the six aims for high-quality health care [7]. Co-operation between services is also highlighted as a main challenge to health care by the IOM, as it was in the present study. Also supporting our results is the research on clinical handover and patient safety, which reveals that co-operation and coordination between involved services are essential for the quality of health care [5].

The indicators designated as recommended or acceptable in the present study are all process measures (i.e., measuring expected mediating factors for health care outcomes). The reservations expressed by the participants in both the focus groups and the expert panels regarding expected confounding factors in the complex referral and care process underline the importance of measuring mediating factors [13, 22, 23]. This finding is in accordance with previous literature on indicators, which asserts that outcome measures are preferred only when it is likely that improvement in the care will lead to significant change in health status or patient evaluation of care [20]. Further, process measures are more sensitive to change and easier to interpret, which is of great importance for facilitating both research and quality improvement efforts [20].

### Strengths and limitations

The existing knowledge about indicators that measure the impact of improved referral information is clearly weak. The RAND/UCLA appropriateness method has become an acknowledged method to define indicators on areas with limited or diverging knowledge by utilising existing knowledge in combination with collective judgments [26, 27]. Further, this method is in line with the thorough preparation of process and outcome measures recommended by the (UK) MRC [24]. However, the method has been criticised for not conveying the patient perspective [61]. In the present study, focus groups representing patients, health professionals and managers were conducted to supplement the limited existing literature and to ensure representation of all stakeholders, as recommended by the framework for developing and assessing the quality of clinical practice guidelines, AGREE II (Appraisal of Guidelines for Research & Evaluation, second version) [62].

A systematic literature review was conducted and presented to the expert panels. However, because there is limited existing literature and the referral and care process is complex, gathering existing knowledge was found to be challenging. Although the search strategy used was assessed to be the most appropriate alternative, there are limitations to the literature review in the present study. The research team found additional relevant literature later in the research process, but this new literature did not introduce new areas or indicators. The lack of more evidence-based studies in the literature review means there are some limitations within the third domain of AGREE II: ‘rigour of development’ [62]. Further, the main body of existing literature found was not within mental health care. However, the combination of a systematic literature review and expert opinion with an agreed standard for the quality of referral information within mental health care, as used in the present study, provides a broader basis for further development of quality indicators and increases content validity in situations with clearly weak evidence bases [61]. The present study included only indicators that were non-specific with regard to condition or diagnosis within the spectrum of mental diseases. For specific conditions, there may be other valid indicators than the ones identified by this study.

### Generalisability

The recommended indicators for measuring the impact of the quality of referral information are based on international literature and focus group interviews representing the relevant perspectives [63]. The results are therefore expected to be valid for mental health services that employ a similar system for the referral process and access to specialised care, as described by Guevara and colleagues [12]. However, participants emphasised that local context can have implications for the interpretation of the data from the indicators. Contextual factors may require adjustments to the definition of the numerator and denominator, and this negatively affects the generalisability of these definitions. The suggested indicators are therefore of interest primarily for improvement efforts-and less for benchmarking-until further exploration of the impact of contextual factors has been conducted. Although the literature review included studies from somatic health care, the participants selected to enrich the evidence base (focus group members) and to assess the qualifications of the indicators (expert panels) were selected for their mental health care expertise. The validity of the findings for services other than mental health is therefore unknown.

## Conclusions

The study revealed a convincing agreement among experts on the expected importance of high quality referral information from primary care to specialised mental health care. The suggested indicators are expected to represent mediating factors affecting a wide range of main goals for health care as defined by the IOM (safe, effective, patient-centred, timely, efficient and equitable) [7]. The risk to health care of not being ‘timely’ is highlighted as particularly relevant when exploring the potential impact of low quality referral information.

We argue that, to enable effective quality improvement, it is necessary to explore underlying mechanisms to understand how outcome indicators can be affected. The present study calls for further inquiry into whether the quality of referral information affects the expected mediating factors for the quality of care and on the predictive value of various types of referral information for the quality of care. This will enable improvements to the suggested definition of high quality referral information by establishing an evidence-based minimum set of information to include in referral requests. Further, it will enable the development of quality improvement interventions tailored to support the underlying mechanisms for achieving high quality mental health care. We recommend that future research emphasise further exploration of mediating factors in the complex referral process, as well as their relevance for patient outcomes, and investigate whether and how contextual factors affect the validity of the suggested indicators.

## Abbreviations

AGREE II: Appraisal of Guidelines for Research & Evaluation, second version
GPs: General Practitioners
IOM: Institute of Medicine
MRC: Medical Research Council
